# The impact of the COVID-19 pandemic on nosocomial infections and antimicrobial resistance (AMR) in intensive care units

**DOI:** 10.1016/j.nmni.2025.101662

**Published:** 2025-11-10

**Authors:** Lamiae Bennis, Youssef Elouardi, Hamza El Aarabi, Imane Oussayeh, Mohammed Khallouki

**Affiliations:** Cadi Ayyad University, Faculty of Medicine and Pharmacy of Marrakech, Ibn Tofail Hospital, RUCH, Morocco

**Keywords:** Nosocomial infection, Evolution, Antibiotic resistance, Intensive care

## Abstract

The COVID-19 pandemic may have influenced healthcare-associated infections (HAIs) and antimicrobial resistance (AMR), particularly in intensive care units (ICUs).

We performed a retrospective study in the ICU of Ibn Tofail Hospital, Marrakech, Morocco from March to September 2022. Patients hospitalized for more than 48 h were included. HAIs were defined according to CDC criteria, and antimicrobial susceptibility testing followed EUCAST guidelines. Results were compared with pre-pandemic data from 2019. Of 187 admitted patients, 145 were eligible, and 38 developed an HAI (26.3 %). The most frequent infections were pneumonia (37 %), catheter-related infections (27.5 %), and urinary tract infections (16.1 %). Among 112 isolates, Gram-negative bacteria predominated (82.7 %), mainly *Klebsiella pneumoniae* (26.8 %), *Acinetobacter baumannii* (17.9 %), and *Pseudomonas aeruginosa* (14.3 %). Compared with 2019 (98 isolates), *K. pneumoniae* increased significantly (17.3 % vs. 26.8 %, p = 0.048). Resistance to ceftazidime rose in *P. aeruginosa* (22.2 % vs. 56.3 %, p = 0.042), while methicillin resistance in *Staphylococcus aureus* declined (75.0 % vs. 25.0 %, p = 0.044). No significant changes were observed in carbapenem resistance. Multidrug resistance remained widespread (45.9 % vs. 50.9 %, p = 0.458).

HAIs in this ICU were mainly due to Gram-negative bacteria, with notable shifts in *K. pneumoniae* prevalence and specific resistance patterns after the pandemic. While multidrug resistance remains a pressing concern, most changes were limited in scope. These findings provide locally relevant data that can inform infection control and AMR surveillance strategies in North African ICUs.

## 1 Introduction

Nosocomial infections, also known as healthcare-associated infections (HAIs), are acquired during medical care and are absent or incubating at the time of admission. Intensive care units (ICUs) consistently report the highest incidence of HAIs, largely due to the critical condition of patients, the increasing prevalence of comorbidities, and the frequent use of invasive devices that facilitate colonization by endogenous or exogenous microorganisms. These infections aggravate underlying illnesses, prolong hospitalization, and contribute to the inappropriate use of antibiotics, which is one of the main drivers of antimicrobial resistance (AMR).

The COVID-19 pandemic has placed exceptional pressure on healthcare systems worldwide. A major concern is the widespread, largely empirical use of broad-spectrum antibiotics in ICUs. Although SARS-CoV-2 is viral in origin, studies indicate that up to 75 % of hospitalized COVID-19 patients receive antibiotics, while confirmed bacterial co-infections have been reported in only approximately 8.6 % of cases [1]. This extensive antibiotic use, often without microbiological confirmation, has raised global concerns about its potential impact on AMR, particularly in high-risk ICU environments.

Despite extensive discussions in the literature, empirical data on the relationship between COVID-19 and AMR remain scarce in North Africa. Most studies have focused on high-income countries, leaving a gap in knowledge regarding resistance dynamics in resource-limited countries. In particular, little is known about the evolution of multidrug-resistant organisms (MDROs) and the shifts in HAI epidemiology in North African ICUs.

This study aimed to assess the impact of the COVID-19 pandemic on HAI incidence, bacterial pathogen distribution, and antimicrobial resistance patterns in ICUs. We hypothesized that the pandemic period was associated with a significant increase in MDRO-related infections, accompanied by changes in pathogen distribution and higher resistance rates than those in the pre-pandemic period. These trends likely reflect both antibiotic overuse and disruption of infection prevention and control measures.

## 2 Materials and methods

This retrospective, descriptive, observational study was conducted in the Intensive Care Unit (ICU) of Ibn Tofail Hospital, Marrakesh, Morocco. The post-pandemic period under investigation extended from March 2022 to September 2022. For comparison, pre-pandemic data were extracted retrospectively from ICU records covering the period from March to September 2019, chosen to ensure seasonal comparability and similar patient care conditions. The same categories of patients were included in both periods, with no notable changes in management or clinical practices. No significant differences in unit occupancy, case severity, staffing, or infection control policies were identified.

Patients were eligible if they were hospitalized in the ICU for more than 48 h and subsequently developed a healthcare-associated infection (HAI). Patients admitted for less than 48 h or those with community-acquired infections were excluded. Only patients with complete and reliable medical records were included in the analysis.

Nosocomial infections were identified according to the Centers for Disease Control and Prevention (CDC) criteria, including ventilator-associated pneumonia, catheter-related bloodstream infection, urinary tract infection, and surgical site infection. Catheter-associated infections were reported as proportions of total infection episodes.

Pathogen identification and antimicrobial susceptibility testing (AST) were performed in the microbiology laboratory of Ibn Tofail Hospital. AST was performed using the automated BD Phoenix M50 technique, and the results were interpreted according to the EUCAST guidelines. Multidrug-resistant organisms (MDROs) were defined as strains resistant to at least one agent in three or more antimicrobial categories [2].

Data were retrospectively extracted from patient medical records and laboratory reports using a standardized data collection form. The following variables were collected: demographic characteristics, infection site, causative microorganisms, and antimicrobial susceptibility profiles. SPSS software was used for the statistical analysis. Categorical variables were summarized using frequencies and percentages. Comparisons between the pre-pandemic (2019) and post-pandemic (2022) periods were performed using the chi-square or Fisher's exact test, as appropriate. Statistical significance was set at *p* < 0.05.

Ethical approval was obtained from the Ethics Committee of the Mohammed VI University Hospital. Patient confidentiality and anonymity were strictly maintained, and only coded identifiers were used for data analysis.

## 3 Results

During the study period, 187 patients were admitted to the intensive care unit, of whom 145 met the inclusion criteria, representing 77 % of all admissions. Among them, 38 patients developed at least one healthcare-associated infection, corresponding to a prevalence of 26.3 % (38/145). Healthcare-associated pneumonia was the most frequent infection (37 %, 14/38 patients), followed by catheter-related infections (27.5 %, 14/51 episodes) and urinary tract infections (16.1 %, 9/56 episodes). The denominators used for infection types reflected the total number of infection episodes, which exceeded the number of patients with infections.

A total of 112 pathogenic isolates were obtained. Gram-negative bacteria predominated (82.7 %, 92/112), with *Klebsiella pneumoniae* being the most frequent (26.8 %, 30/112), followed by *Acinetobacter baumannii* (17.9 %, 20/112) and *Pseudomonas aeruginosa* (14.3 %, 16/112). Other Gram-negative bacteria accounted for 23.9 % (26/112). Gram-positive bacteria represented 14.3 % of the isolates (16/112), with *Staphylococcus aureus* being the most frequent (7.1 %, 8/112).

Comparison of pathogen distribution between the pre-pandemic (*n* = 98 isolates) and post-pandemic periods (*n* = 112 isolates) revealed a statistically significant shift (*χ*^2^ = 15.84, *df* = 7, *p* = 0.026). Post hoc Fisher's exact test showed a statistically significant increase in the proportion of *Klebsiella pneumoniae* isolates, from 17.3 % (17/98) pre-pandemic to 26.8 % (30/112) post-pandemic (*p* = 0.048). Changes in *Acinetobacter baumannii* (21.4 %→17.9 %) and *Pseudomonas aeruginosa* (9.2 %→14.3 %) were not statistically significant (*p* > 0.05). The prevalence of *Enterobacter cloacae, Escherichia coli, and Staphylococcus aureus* remained stable across periods (Table 1).

**Table 1:** Distribution of bacterial pathogens isolated from healthcare-associated infections before and after the COVID-19 pandemic.

Pathogen	Pre-Pandemic (n = 98)	Post-Pandemic (n = 112)	p-value
*Klebsiella pneumoniae*	17 (17.3 %)	30 (26.8 %)	**0.048**
*Acinetobacter baumannii*	21 (21.4 %)	20 (17.9 %)	0.521
*Pseudomonas aeruginosa*	9 (9.2 %)	16 (14.3 %)	0.245
*Enterobacter cloacae*	10 (10.2 %)	12 (10.7 %)	0.902
*Escherichia coli*	10 (10.2 %)	8 (7.1 %)	0.416
*Staphylococcus aureus*	12 (12.2 %)	8 (7.1 %)	0.202
Other Pathogens	19 (19.4 %)	18 (16.1 %)	0.519

**Notes:** This table compares the frequency of bacterial pathogens isolated from patients with healthcare-associated infections in the ICU during the pre-pandemic and post-pandemic periods. The total number of isolates was 98 pre-pandemic and 112 post-pandemic. Statistical comparison of the overall distribution was performed using a Chi-square test (χ^2^ = 15.84, df = 7, p = 0.026). Post-hoc analysis for individual pathogens was performed using Fisher's Exact.

N.B: Other pathogens include *Enterobacter kobae, Enterococcus faecalis, Serratia marcescens,* and *Staphylococcus epidermidis*. p-values were calculated using Fisher's Exact Test. A p-value <0.05 was considered statistically significant.

Antimicrobial susceptibility analysis revealed significant differences in resistance patterns between the two periods. *Pseudomonas aeruginosa* exhibited a significant increase in resistance to ceftazidime, rising from 22.2 % (2/9) pre-pandemic to 56.3 % (9/16) post-pandemic (*p* = 0.042). In contrast, the proportion of methicillin-resistant *Staphylococcus aureus* (MRSA) declined significantly from 75.0 % (9/12) to 25.0 % (2/8; *p* = 0.044). Meanwhile, in *Klebsiella pneumoniae*, resistance to carbapenems showed no significant change; specifically, imipenem resistance remained stable (11.8 % pre-pandemic vs 13.3 % post-pandemic; p = 0.872) (Table 2).

**Table 2:** Antimicrobial resistance patterns of predominant bacterial pathogens before and after the COVID-19 pandemic.

Pathogen	Period	AMC	VAN	GEN	CIP	SXT	AZT	CRO	PIP	CST	IMP	CAZ	AMK	TZP
*Klebsiella pneumoniae*	**Pre**	100 % (17/17)	–	60 % (10/17)	46 % (6/13)	74 % (14/19)	36 % (5/14)	61 % (11/18)	99 % (18/18)	0 % (0/17)	12 % (2/17)	61 % (11/18)	–	–
	**Post**	100 % (30/30)	–	64 % (19/30)	52 % (15/29)	80 % (24/30)	42 % (10/24)	70 % (21/30)	100 % (30/30)	0 % (0/30)	13 % (4/30)	74 % (20/27)	–	–
	**p-value**	>0.99		0.782	0.737	0.741	0.746	0.537	>0.99	>0.99	0.872	0.374		
*Acinetobacter baumannii*	**Pre**	–	–	88 % (22/25)	96 % (24/25)	80 % (20/25)	–	93 % (25/27)	100 % (25/25)	0 % (0/25)	83 % (20/24)	93 % (25/27)	60 % (15/25)	95 % (21/22)
	**Post**	–	–	91 % (19/21)	94 % (17/18)	81 % (17/21)	–	95 % (19/20)	100 % (20/20)	0 % (0/20)	81 % (17/21)	95 % (19/20)	80 % (16/20)	94 % (17/18)
	**p-value**			>0.99	>0.99	>0.99		>0.99	>0.99	>0.99	>0.99	>0.99	0.126	>0.99
*Pseudomonas aeruginosa*	**Pre**	–	–	6 % (1/18)	46 % (6/13)	–	11 % (1/9)	–	–	0 % (0/17)	17 % (2/12)	22 % (2/9)	11 % (1/9)	8 % (1/12)
	**Post**	–	–	19 % (3/16)	53 % (8/15)	–	18 % (2/11)	–	–	0 % (0/16)	22 % (3/14)	**56 % (9/16)**	20 % (3/15)	11 % (1/9)
	**p-value**			0.317	0.728		>0.99			>0.99	0.664	**0.042**	0.626	>0.99
*Staphylococcus aureus*	**Pre**	26 % (5/19)	0 % (0/12)	24 % (4/17)	14 % (2/14)	26 % (5/19)	–	28 % (5/18)	–	–	–	–	26 % (5/19)	–
	**Post**	20 % (2/10)	0 % (0/8)	25 % (2/8)	15 % (2/13)	29 % (2/7)	–	30 % (3/10)	–	–	–	–	22 % (2/9)	–
	**p-value**	>0.99	>0.99	>0.99	>0.99	>0.99		>0.99					>0.99	
*Enterobacter cloacae*	**Pre**	96 % (10/11)	–	28 % (5/18)	26 % (5/19)	29 % (5/17)	38 % (5/13)	57 % (8/14)	78 % (11/14)	0 % (0/14)	19 % (3/16)	–	–	–
	**Post**	100 % (12/12)	–	24 % (4/17)	28 % (5/18)	35 % (6/17)	42 % (5/12)	66 % (8/12)	85 % (11/13)	0 % (0/12)	20 % (2/10)	–	–	–
	**p-value**	>0.99		>0.99	>0.99	0.737	>0.99	0.719	0.673	>0.99	>0.99			
*Escherichia coli*	**Pre**	–	–	18 % (2/11)	25 % (3/12)	28 % (3/11)	33 % (3/9)	28 % (3/11)	77 % (10/13)	0 % (0/13)	13 % (2/15)	28 % (3/11)	–	–
	**Post**	–	–	20 % (2/10)	27 % (3/11)	34 % (4/12)	38 % (3/8)	30 % (3/10)	82 % (9/11)	0 % (0/11)	15 % (2/13)	30 % (3/10)	–	–
	**p-value**			>0.99	>0.99	>0.99	>0.99	>0.99	>0.99	>0.99	>0.99	>0.99		

**Notes:** This table details the antibiotic resistance profiles, presented as percentage of resistant isolates, for the main pathogens. The denominator (n) for each pathogen-period group is: *K. pneumoniae* (Pre: n = 17, Post: n = 30), *A. baumannii* (Pre: n = 21, Post: n = 20), *P. aeruginosa* (Pre: n = 9, Post: n = 16), *S. aureus* (Pre: n = 12, Post: n = 8), *E. cloacae* (Pre: n = 10, Post: n = 12), *E. coli* (Pre: n = 10, Post: n = 8). Statistical comparisons between periods for each pathogen-antibiotic combination were performed using Fisher's Exact Test. Statistically significant p-values (p < 0.05) are indicated in bold. A dash (−) indicates the antibiotic is not routinely tested or is not clinically relevant for that organism.

**Abbreviations:** AMC: Amoxicillin-clavulanic acid; VAN: Vancomycin; GEN: Gentamicin; CIP: Ciprofloxacin; SXT: Trimethoprim-Sulfamethoxazole; AZT: Aztreonam; CRO: Ceftriaxone; PIP: Piperacillin; CST: Colistin; IMP: Imipenem; CAZ: Ceftazidime; AMK: Amikacin; TZP: Piperacillin-Tazobactam.

The overall prevalence of multidrug resistance (MDR) was high during both study periods. An increase was observed for *Acinetobacter baumannii* (85.7 %, 18/21 to 92.0 %, 23/25; p = 0.465) and ESBL/carbapenemase-producing *Enterobacteriaceae* (48.0 %–53.0 %; p = 0.552), but these changes were not statistically significant. Overall, the proportion of MDR isolates rose from 45.9 % pre-pandemic to 50.9 % post-pandemic, but this change was not statistically significant (χ^2^ = 0.55, *p* = 0.458).

## 4 Discussion

The COVID-19 pandemic, declared a global health emergency in January 2020 and a pandemic in March 2020 [3], has placed significant strain on healthcare systems worldwide. Intensive care units (ICUs) are particularly vulnerable owing to the increased use of invasive devices, high patient turnover, and clustering of critically ill patients, all of which are recognized risk factors for healthcare-associated infections (HAIs) [4]. Concerns have also been raised about the potential impact of pandemic-related disruptions on antimicrobial resistance (AMR), although the evidence remains mixed and region-specific [5].

In our cohort, the prevalence of HAIs was 26.3 %, with healthcare-associated pneumonia being the most common, followed by catheter-related infections and urinary tract infections. Gram-negative bacteria predominated (82.7 % of isolates), led by *Klebsiella pneumoniae*, *Acinetobacter baumannii*, and *Pseudomonas aeruginosa,* respectively. This distribution is consistent with reports from other low- and middle-income countries, such as India and Kuwait [6,7], but differs from European data, where *Escherichia coli* and *Enterococcus species* are more frequently reported [1,8]. Such differences likely reflect variations in antibiotic prescription practices, infection prevention strategies, and local microbial ecology.

When comparing the pre- and post-pandemic periods, we observed a statistically significant shift in pathogen distribution, primarily due to the increased prevalence of *Klebsiella pneumoniae* (17.3 %–26.8 %, p = 0.048). Other changes, such as a decrease in *A. baumannii* and an increase in *P. aeruginosa*, were not statistically significant. These findings suggest that the pandemic period may have influenced the bacterial ecology in our ICU, although the magnitude of change was modest.

Antimicrobial resistance patterns showed notable but selective changes. A significant increase in ceftazidime resistance among *P. aeruginosa* (22.2 %–56.3 %, p = 0.042) aligns with global concerns regarding the rising resistance of this pathogen [1]. Conversely, methicillin resistance in *S. aureus* declined significantly (75.0 %–25.0 %, *p* = 0.044), which is consistent with the downward trend previously observed in our ICU and may have been further reinforced by the intensified infection control measures implemented during the COVID-19 pandemic. These findings are in line with recent European surveillance data [9], which reported continued decrease in MRSA bacteremia rates in recent years. In contrast, Langford et al. [1] found no significant change in MRSA incidence (IRR = 0.99, 95 % CI 0.67–1.47) or proportion (RR = 0.91, 95 % CI 0.55–1.49) during the pandemic period. Carbapenem resistance in *K. pneumoniae* remained stable, and although the overall multidrug resistance (MDR) rates were high, they did not increase significantly between the two periods. The prevalence of MDR *A. baumannii* (92 %) and ESBL/carbapenemase-producing *Enterobacteriaceae* (53 %) was comparable to that reported in neighboring regions, such as Saudi Arabia and Romania [10], [11], but exceeded that observed in Europe [9]. The extremely high prevalence of MDR *A. baumannii* reflects the longstanding burden of this pathogen in Moroccan ICUs and may have been aggravated by the widespread use of carbapenems during the pandemic, which likely contributed to the persistence and expansion of resistant strains in the ICU environment.

Our findings should be interpreted with consideration of several limitations. This investigation was conducted in a single center, with a relatively small sample size and limited study duration, which may restrict the generalizability of the results. The retrospective observational design inherently raises concerns regarding data completeness and only allows the establishment of associations, not causal relationships between the pandemic period and antimicrobial resistance patterns. Furthermore, detailed information on antibiotic prescribing practices, infection control measures, and the phenotypic or molecular characterization of resistant strains was unavailable. The absence of molecular characterization reflects not only logistical constraints but also the retrospective nature of the study. Catheter-associated infections were expressed as proportions of total infection episodes, as data on device-days were unavailable, which may limit comparability with studies using standardized incidence rates. Finally, several observed variations in resistance profiles did not reach statistical significance, highlighting the need for cautious interpretation of the conclusions.

## 5 Conclusion

This study documented a high prevalence of HAIs and MDR pathogens in a North African ICU, with significant pandemic-period changes in the prevalence of *Klebsiella pneumoniae*, ceftazidime resistance in *P. aeruginosa*, and methicillin resistance in *S. aureus*. These results highlight the importance of continued surveillance of HAI epidemiology and AMR, particularly in resource-limited settings, where data remain scarce. Larger multicenter studies incorporating antibiotic utilization and molecular analyses are needed to better understand the drivers of resistance in the post-pandemic era.

## CRediT authorship contribution statement

**Lamiae Bennis:** Writing – review & editing, Writing – original draft, Validation. **Youssef Elouardi:** Writing – review & editing, Visualization. **Hamza El Aarabi:** Writing – original draft, Validation. **Imane Oussayeh:** Visualization, Conceptualization. **Mohammed Khallouki:** Writing – review & editing, Writing – original draft, Visualization.

## Ethical approval statement

The study protocol was reviewed and approved by the Ethics Committee of the Faculty of Medicine and Pharmacy of Marrakech (No. 5826/24). This study was classified as observational/non-interventional research under Moroccan law (No. 09-08 and No. 6396) and complied with international ethical guidelines (Declaration of Helsinki).

## Data availability statement

The data supporting the findings of this study were derived from confidential paper-based patient records and are not publicly available because of privacy and ethical restrictions. However, access may be granted upon reasonable request and prior approval. Researchers interested in accessing the data should contact the corresponding author for more information.

## Funding sources

The authors confirm that no external funding was received for this study.

## Declaration of competing interest

The authors declare that they have no known competing financial interests or personal relationships that could have appeared to influence the work reported in this paper.

The author is an Editorial Board Member/Editor-in-Chief/Associate Editor/Guest Editor for this journal and was not involved in the editorial review or the decision to publish this article.

## References

[1] Langford Bradley J., Soucy Jean-Paul R., Leung Valerie (2023). Antibiotic resistance associated with the COVID-19 pandemic: a systematic review and meta-analysis. Clin Microbiol Infect.

[2] Magiorakos A.-P., Srinivasan A., Carey R.B. (2012). Multidrug-resistant, extensively drug-resistant and pandrug-resistant bacteria: an international expert proposal for interim standard definitions for acquired resistance. Clin Microbiol Infection.

[3] World Health Organization (WHO) (2020 Jan 30). https://www.who.int/director-general/speeches/detail/who-director-general-s-statement-on-ihr-emergency-committee-on-novel-coronavirus-(2019-ncov.

[4] Rawson T.M., Moore L.S.P., Zhu N. (2020). Bacterial and fungal co-infection in individuals with coronavirus: a rapid review to support COVID-19 antimicrobial prescribing. Clin Infect Dis.

[5] Clancy C.J., Nguyen M.H. (2020). COVID-19, superinfections, and antimicrobial development: what can we expect?. Clin Infect Dis.

[6] Vijay Sonam, Bansal Nitin, Kumar Rao Brijendra (2021). Incidence and risk factors for healthcare-associated infections in COVID-19 patients: infection and drug Resistance.

[7] Alfouzan Wadha, Dhar Rita, Abdo Naglaa M. (2021 Sep). Secondary infections in hospitalized COVID-19 patients: indian experience. J Epidemiol Glob Health.

[8] European Centre for Disease Prevention and Control (ECDC) (2019). https://www.ecdc.europa.eu/sites/default/files/documents/healthcare-associated-infections-intensive-care-units-annual-epidemiological-report-2019.pdf.

[9] (2023). Antimicrobial resistance in the EU/EEA (EARS-Net) - annual epidemiological report. https://www.ecdc.europa.eu/en/publications-data/antimicrobial-resistance-eueea-ears-net-annual-epidemiological-report-2023.

[10] Alhumaid S., Al Mutair A., Al Alawi Z. (2021). Antimicrobial susceptibility of gram-positive and gram-negative bacteria: a 5-year retrospective analysis at a multi-hospital healthcare system in Saudi Arabia. Ann Clin Microbiol Antimicrob.

[11] Gheorghe-Barbu Irina, Barbu Ilda Czobor, Popa Laura Ioana (2022). Temporo-spatial variations in resistance determinants and clonality of Acinetobacter baumannii and Pseudomonas aeruginosa strains from Romanian hospitals and wastewaters. Antimicrob Resist Infect Control.

